# Physiological Functions of Thiol Peroxidases (Gpx1 and Prdx2) during *Xenopus laevis* Embryonic Development

**DOI:** 10.3390/antiox10101636

**Published:** 2021-10-17

**Authors:** Hongchan Lee, Na Young Lee, Youni Kim, Hong-Seok Choi, Tayaba Ismail, Hong-Yeoul Ryu, Dong-Hyung Cho, Zae Young Ryoo, Dong-Seok Lee, Taeg Kyu Kwon, Tae Joo Park, Taejoon Kwon, Hyun-Shik Lee

**Affiliations:** 1 BK21 FOUR KNU Creative BioResearch Group, School of Life Sciences, Kyungpook National University, Daegu 41566, Korea; leehongchan@hanmail.net (H.L.); ny0726@knu.ac.kr (N.Y.L.); poloqq@knu.ac.kr (Y.K.); hschoi9784@knu.ac.kr (H.-S.C.); tayaba@knu.ac.kr (T.I.); rhr4757@knu.ac.kr (H.-Y.R.); dhcho@knu.ac.kr (D.-H.C.); jaewoong64@knu.ac.kr (Z.Y.R.); lee1@knu.ac.kr (D.-S.L.); 2 Department of Immunology, School of Medicine, Keimyung University, Daegu 41566, Korea; kwontk@dsmc.or.kr; 3 Department of Biological Sciences, College of Information-Bio Convergence, Ulsan National Institute of Science and Technology (UNIST), Ulsan 44919, Korea; parktj@unist.ac.kr; 4 Department of Biomedical Engineering, College of Information-Bio Convergence, Ulsan National Institute of Science and Technology (UNIST), Ulsan 44919, Korea

**Keywords:** Gpx1, Prdx2, embryogenesis, eye development, lens detachment, morpholinos, VBI

## Abstract

Glutathione peroxidase 1 (Gpx1) and peroxiredoxin 2 (Prdx2) belong to the thiol peroxidase family of antioxidants, and have been studied for their antioxidant functions and roles in cancers. However, the physiological significance of Gpx1 and Prdx2 during vertebrate embryogenesis are lacking. Currently, we investigated the functional roles of Gpx1 and Prdx2 during vertebrate embryogenesis using *Xenopus laevis* as a vertebrate model. Our investigations revealed the zygotic nature of *gpx1* having its localization in the eye region of developing embryos, whereas *prdx2* exhibited a maternal nature and were localized in embryonic ventral blood islands. Furthermore, the *gpx1*-morphants exhibited malformed eyes with incompletely detached lenses. However, the depletion of *prdx2* has not established its involvement with embryogenesis. A molecular analysis of *gpx1*-depleted embryos revealed the perturbed expression of a *cryba1*-lens-specific marker and also exhibited reactive oxygen species (ROS) accumulation in the eye regions of *gpx1*-morphants. Additionally, transcriptomics analysis of *gpx1*-knockout embryos demonstrated the involvement of Wnt, cadherin, and integrin signaling pathways in the development of malformed eyes. Conclusively, our findings indicate the association of *gpx1* with a complex network of embryonic developmental pathways and ROS responses, but detailed investigation is a prerequisite in order to pinpoint the mechanistic details of these interactions.

## 1. Introduction

Aerobic metabolism results in cellular energy production along with the generation of a toxic oxygen intermediate termed “reactive oxygen species” (ROS), which in excess amounts causes cellular homeostasis disturbance [1]. Among the different ROS, peroxides (e.g., H_2_O_2_) are generated by a diverse variety of metabolic processes, such as superoxide anion dismutation, or as oxidase byproducts [2]. Organic peroxides are also produced in the cells by cyclooxygenase and lipoxygenase actions or due to polyunsaturated fatty acid oxidation [2]. ROS are important signaling molecules at physiological or low levels in the cells, activating/deactivating important signaling pathways [3], and they play significant roles in embryonic development [4]. However, excess amounts of ROS damage cells, and the critical balance of ROS is crucial for the body’s proper functioning and embryonic development [5,6]. The homeostatic ROS balance is maintained through various enzymatic and non-enzymatic quenchers [7].

Thiol peroxidases play an essential role in the intracellular metabolism of peroxides [8] and comprise the glutathione peroxidase (GPx) and peroxiredoxin (Prdx) families of enzymatic proteins [9]. GPxs and Prdxs are mechanistically distinct from each other, but they share overlapping substrate specificities [9]. Both depend on intracellular electron donors for their peroxidase activity against H_2_O_2_ and organic peroxides [8]. GPxs containing selenocysteine at their active site depend on glutathione to reduce hydro-peroxides [10]. Mammalian cells contain at least four types of GPxs, but GPx1 is the only glutathione peroxidase present in the erythrocytes [11]. Prdxs are a large and highly conserved family of peroxiredoxins that catalyze the reduction of peroxides by conserved cysteine residue, termed peroxidatic cysteine, serving as the oxidation site for peroxides [12,13]. In mammals, there are six types of Prdxs, among which only Prdx2 is abundant in erythrocytes [14,15]. They play an essential role in the antioxidant defense of erythrocytes, along with catalase. In this way, these two thiol peroxidases, i.e., GPx1 and Prdx2, share a similarity in their occurrence. Studies have shown that Gpx1 overexpression leads to enhanced protection against oxidative stress, but Gpx1-overexpressing mice exhibit obesity, insulin resistance, hyperinsulinemia, and hyperglycemia [16]. Gpx1 has also been known for its significant roles in cancer [17,18]. Reduced mRNA expression and the DNA promoter methylation of Gpx1 have been detected in gastric cancer cell lines [19]. Furthermore, Gpx1 significance has been observed in breast cancer [20]. It is reported that mice lacking Prdx2 develop hemolytic anemia [21], but they possess fully functional catalase and glutathione peroxidase [14]. This implies the specific and non-redundant functions of erythrocyte Prdx2 in peroxide removal and the protection of erythrocytes against oxidative damage [14]. Prdx2 has been reported to have a high expression level in various cancers, including in lung, colorectal, and gastric cancers [22]. Moreover, Prdx2 has been reported to be involved in the progression of arthrosclerosis [23].

The critical balance of ROS is indispensable for embryonic development, and the members of thiol peroxidases have been studied for their essential roles in early vertebrate embryogenesis, for example, Prdx1 plays a crucial role in pronephros development during embryogenesis [24], and Prdx5 has been found to control vertebrate ciliogenesis [25]. In addition to Prdxs, GPx3 is involved in posterior development during vertebrate embryonic development [26]. Based on the pronounced roles of the members of thiol peroxidases during vertebrate embryonic development, we sought to investigate the physiological significance of erythrocyte’s thiol peroxidases, i.e., GPx1 and Prdx2, during early embryogenesis.

In this study, we employed *Xenopus laevis* as a vertebrate model to investigate the developmental significance of Gpx1 and Prdx2. The spatiotemporal expression of *gpx1* indicated its zygotic nature, exhibiting its localization in the eye region, particularly in the developing lenses, whereas *prdx2* was expressed as a maternal gene exhibiting its expression in the embryonic ventral blood islands (VBI). Loss-of-function studies using *gpx1* and *prdx2* morpholino oligonucleotide (MOs) revealed incompletely detached lenses with a smaller diameter, perturbed expression of lens-specific markers, and increased ROS levels in *gpx1*-morphant embryos, but the knockdown of *prdx2* did not induce any significant malformation during *Xenopus* embryogenesis. *gpx1* depletion led to interference with the Wnt and cadherin signaling pathways associated with normal eye development. These findings clearly showed the prerequisite functions of *gpx1* in vertebrate eye development, but no particular significance of *prdx2* was observed during *Xenopus* embryogenesis.

## 2. Materials and Methods

### 2.1. Xenopus Growth Conditions and In Vitro Fertilization

The Korean *Xenopus* Resource Center for Research provided the adult *Xenopus laevis,* and they were kept at 18 °C under a 12 h photoperiod in containers recommended by the Institutional Review Board of the Kyungpook National University, Daegu, Korea. The *Xenopus* females were injected with 1000 IU of human chorionic gonadotropins into the dorsal lymph sac in the evening before the experiment for ovulation induction. The following morning, the females were transferred to 1× high salt solution for egg harvesting. For fertilization, the male *Xenopus* were kept in 1× benzocaine solution for 5–15 min and were then sacrificed to isolate the testis. Isolated testes were kept in 1× MBS at 4 °C. The eggs were washed with 0.1× MBS thrice and then fertilized with sperm suspension solution derived from the isolated testes. After successful fertilization, the embryos were de-jellied by swirling in 2% L-cysteine and were then washed with 0.5× MBS five times. The unfertilized and dead eggs were removed by observing under a light stereomicroscope. The healthy and fertilized eggs were cultured at 15−18 °C in 0.5× MBS containing 2% Ficoll 400 (GE Healthcare, Little Chalfont, UK).

### 2.2. Plasmids and mRNA Synthesis

The cDNA was synthesized from the total RNA of the tailbud stage embryos. Based on the *gpx1* (Accession no. NM_001095427) and *prdx2* (Accession no. NM_001091945) sequence in NCBI and Xenbase, primers were designed to clone *gpx1* and *prdx2*. Flag-tagged *gpx1* mRNA and *prdx2* mRNA were generated by PCR. Plasmids were constructed using the T-easy vector and pCS107 vector for *gpx1* and *prdx2,* respectively, and the mRNAs were synthesized using SP6 mMessage mMachine kit (Ambion, Woodward Street, Austin, TX, USA).

### 2.3. Morpholino Oligonucleotides Design and Xenopus Embryos Microinjection

The *gpx1* MO (Gene Tools, Philomath, USA) used was 25 nucleotides and was designed to bind the translation initiation region of the *gpx1* mRNA; the sequence of the *X. laevis gpx1* translation-blocking MO was 5′-ATGCGTTCAGCTATGGTTTCTCGCA-3′. The splice-blocking *gpx1* MOs were as follows, 5′-CAGTTGTTCCCTTGTCACTCACCTG-3′. The *prdx2* morpholino (MO) (Gene Tools) used was 25 nucleotides long and had the following base composition, 5′-AATTTCACATC (ATG) GCGTGTCCTGTG-3′. The MOs were injected into the animal pole of two-cell stage embryos, and green fluorescent proteins mRNAs were coinjected as a lineage tracer.

The HyPer-cyto gene was obtained from pHyPer-cyto (Evrogen, Moscow, Russia) and was subcloned into the pCS2+ vector. HyPer-pCS2+ was linearized using the NotI restriction enzyme. The SP6 mMessage mMachine kit was used to synthesize mRNA (Ambion) [26].

### 2.4. Whole-Mount In Situ Hybridization (WISH)

*gpx1* and *prdx2* MOs were injected into one or both blastomeres of two cells staged with *Xenopus* embryos individually, and the MO-injected embryos at the desired stage were fixed in MEMFA (4% paraformaldehyde, 0.1-M MOPS (pH7.4), 1-mM MgSO4, 2-mM EGTA) overnight at 4 °C and then dehydrated before storage in 100% methanol at −20 ℃. To prepare the antisense DIG-labeling probes of *hba3* (Accession no. NM_001086328), *mpo* (Accession no. NM_001087639), and *cryba1* (Accession no. NM_001094493), DNA templates were linearized using appropriate restriction enzymes. The probes were synthesized using SP6 or T7 RNA polymerase (Ambion) and were detected using an alkaline phosphatase-labeled anti-digoxigenin antibody (1:1000, Roche, Basel, Switzerland) and NBT/BCIP (nitro blue tetrazolium/5-bromo-4-chloro-3indolyl phosphate) [27].

### 2.5. RT-PCR (Reverse Transcription–Polymerase Chain Reaction)

The total RNA was extracted from *Xenopus laevis* embryos using Isol-RNA lysis reagent (5 Prime GmbH, Hilden, Germany). cDNA was synthesized using a PrimeScript™ first-strand cDNA synthesis kit (Takara, Kusatsu, Japan) using the total RNA extracted from *Xenopus* embryos. PCR was conducted using customized primers, and the products were loaded on 1% agarose gels. The images were captured using WiseCapture I-1000 (Daihan Scientific, Wonju, Korea).

### 2.6. Transcriptomic Analysis

The RNA sequencing library was constructed using the total RNA extracted from each sample using polyA enrichment, according to the manufacturer’s instructions (Illumina, San Diego, CA, USA). To estimate the mRNA abundance, *X. laevis* cDNA sequence reads were mapped from the genome project consortium using bwa (version 0.7.15), and then, edgeR (version 3.20.7) was used to analyze differentially expressed genes (DEGs). Genes with > 4-fold change and a false discovery rate (FDR) < 0.01 in the exact test were considered to show a significant differential expression. Over-represented biological processes in these DEGs were tested using Fisher’s test provided by the PANTHER database (released 20171205) with human orthologous genes based on best hits using BLASTP search. Raw data for RNA-seq are available in the ENA database (accession numbers: ERS4819529 and ER4819530 for the control and ERS4819534 and ERS4819535 for the *gpx1* MOs) [28].

### 2.7. Terminal Deoxynucleotidal Transferase dUTP Nick End Labeling (TUNEL) Staining

*Xenopus* embryos were fixed for 24 h in MEMFA (4% paraformaldehyde, 0.1-M MOPS pH 7.4, 1-mM MgSO4, and 2-mM EGTA) at 4 °C and were bleached using bleaching solution (3% H_2_O_2_, 5% formamide, and 5X SSC) after PBS washing. The embryos were processed for TUNEL [26,27].

### 2.8. In Vivo Imaging of ROS

HyPer cyto (HyPer) was used to detect H_2_O_2_ related ROS. HyPer mRNAs (10 ng) were injected into the D.1.2. blastomere of 16 cell stage embryos. Images were taken using live anesthetized embryos using 1:1000 diluted benzocaine and an Olympus FV1200 confocal microscope. Images were analyzed by ImageJ (NIH; http://imagej.nih.gov; accessed on 10 June 2021) [26].

### 2.9. Statistical Analysis

Data from WISH and RT-PCR were analyzed using ImageJ software (NIH; http://imagej.nih.gov; accessed on 10 June 2021). The results were presented as the mean ± standard error from at least three independent experiments and were interpreted by paired and unpaired *t*-test using GraphPad Prism 7 software (GraphPad Software Inc., La Jolla, CA, USA). *P*-values of less than 0.05 were considered statistically significant and were represented by an asterisk (*).

## 3. Results

### 3.1. Spatiotemporal Expression Pattern of gpx1 and prdx2 during Xenopus Embryogenesis

**Figure 1:**
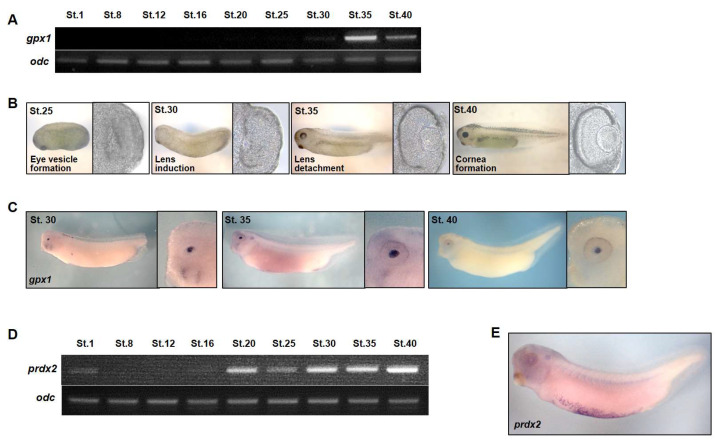
Spatiotemporal expression pattern of *gpx1* and *prdx2* during *Xenopus* embryogenesis. (**A**) The transcriptase-polymerase chain reaction (RT-PCR) analysis indicated the zygotic nature of *gpx1* during *Xenopus* embryonic development. The temporal expression of *gpx1* started at NF. St. 30 of embryogenesis and proceeded until NF. St. 40. The highest expression level was observed at NF. St. 35 of *Xenopus* embryogenesis. Ornithine decarboxylase (*odc*) was used as an internal loading control. (**B**) The general scheme for *Xenopus* eye development in the tadpole stage indicates eye vesicle formation at NF. St. 25, followed by lens induction at NF. St. 30., while lens detachment begins at NF. St. 35, leading to cornea formation at NF. St 40 of *Xenopus* embryonic development. (**C**) The spatial expression of *gpx1* as analyzed by whole-mount in situ hybridization (WISH) indicated its specific expression in the lens after eye vesicle formation at NF. St 30 embryonic development with an increased expression level in the lens region at NF. St. 35 and it proceeded until NF. St. 40 of *Xenopus* embryogenesis. (**D**) RT-PCR analysis revealed the maternal nature of *prdx2* during *Xenopus* embryogenesis. The highest level of *prdx2* expression was observed at the late neurula and tailbud stages of embryogenesis. Ornithine decarboxylase (*odc*) was used as the internal loading control. (**E**) WISH analysis indicated the localization of *prdx2* in embryonic VBIs during *Xenopus* embryonic development.

To investigate the specific roles of *gpx1* and *prdx2* during embryogenesis, we determined their expression pattern by conducting RT-PCR and WISH analyses. RT-PCR indicated the zygotic nature of *gpx1* as it exhibited its expression at the tadpole stage (Nieuwkoop and Faber; St. 30) of *Xenopus* embryonic development (Figure 1A) and maintained its expression until NF. St. 40 of embryogenesis (Figure 1A). The highest expression level was observed at NF. St. 35 of the embryonic development (Figure 1A). The temporal *gpx1* expression at NF. St. 30 of embryogenesis made us speculate about the involvement of *gpx1* in eye development during *Xenopus* embryogenesis because optic vesicle formation starts at the NF. St. 25 of embryonic development, followed by lens induction at NF. St. 30, lens detachment at NF. St. 35, and finally cornea formation at NF. St. 40 of embryonic development (Figure 1B). To evaluate our speculation, we conducted a WISH analysis to determine the spatial expression of *gpx1* during *Xenopus* embryogenesis. Our results showed that *gpx1* is expressed explicitly in the lens region of developing embryos at NF. St. 30 of embryogenesis, and its expression is sustained until NF. St. 40 in the lens region of developing embryos (Figure 1C). Based on the spatiotemporal expression of *gpx1*, it is evident that it may have critical roles during the eye development stage of embryogenesis.

In case of *prdx2*, we examined the spatiotemporal pattern of *prdx2,* and the temporal expression pattern of *prdx2* indicated the maternal nature of the gene as it exhibited its expression from the initial stages of embryogenesis until stage 40 of embryonic development (Figure 1D). The enhanced expression of *prdx2* was observed at NF. St 20 (late neurula) of *Xenopus* embryonic development (Figure 1D). The spatial expression of *prdx2* as investigated by the WISH analysis indicated its localization in the embryonic VBI (Figure 1E), and it was in the same position as where the posterior VBI and myeloid cells are distributed. Based on the spatial expression of *prdx2*, we speculated *prdx2* involvement in embryonic blood cell development.

### 3.2. gpx1 Knockdown Leads to Malformed Eyes, but prdx2 Morphant Embryos Exhibited No Specific Malformation during Xenopus Embryonic Development

**Figure 2:**
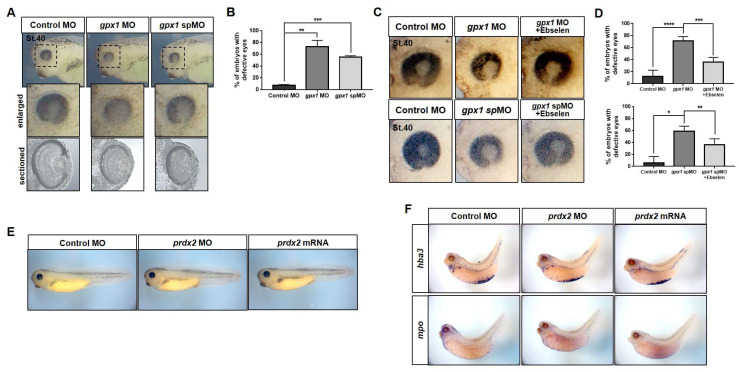
*gpx1* loss-of-function results in malformed eyes with incompletely detached lenses, while *prdx2* morphant embryos have no phenotypic malformations. (**A**) Two types of *gpx1* morpholino oligonucleotides (MOs), i.e., translational blocking MO (*gpx1* MO; 20 ng) and splicing blocking MO (*gpx1* spMO; 5 ng), were microinjected into animal poles of two-cell stage *Xenopus* embryos, and then the embryos were fixed at NF. St. 40. Knockdown of *gpx1* in both groups of MOs resulted in malformed eyes, as shown in the enlarged images. Additionally, the cross-sectional analysis indicated the incompletely detached lens in both groups of *gpx1* morphants. (**B**) The statistical analysis of *gpx1* morphants indicated that more than 70% of the embryos exhibited malformed eyes in the case of *gpx1* MO microinjection, while more than 50% of embryos showed eye malformations in the case of *gpx1* spMO injection compared with the control embryos. ** *p* ≤ 0.01, *** *p* ≤ 0.001. (**C**) Rescue experiments were conducted by transferring the *gpx1* morpholino injected embryos to 0.5-μΜ ebselen, a Gpx mimic, and then fixed at NF. St. 40. The eye malformations observed in both groups of *gpx1*-depleted embryos were effectively recovered after ebselen treatment. (**D**) A graphical representation of rescued embryos showed that more than 30% of embryos recovered the malformed eyes observed due to *gpx1* MO and *gpx1* spMO microinjection after ebselen treatment. * *p* ≤ 0.05 ** *p* ≤ 0.01, *** *p* ≤ 0.001, **** *p* ≤ 0.0001. (**E**) *prdx2* MO (40 ng) and wildtype *prdx2* mRNA (1 ng) were microinjected into the two cell stage developing embryos and then the embryos were fixed at NF. St. 40. The embryos developed normally after *prdx2* MO injection and there were no phenotypic differences between *prdx2* morphant embryos and embryo injected with wildtype *prdx2* mRNA. Both group of embryos were morphologically similar to the control MO injected embryos. (**F**) *Xenopus* embryos were injected with *prdx2* MO and were processed for WISH analysis using blood cell markers, i.e., *hba3* and *mpo*. The knockdown of *prdx2* did not affect the expression pattern of the blood cell-specific markers.

To evaluate our speculation of *gpx1* involvement in eye development and the *prdx2* association with embryonic blood cell development during embryogenesis, we performed loss-of-function studies using antisense *gpx1* and *prdx2* MOs. For *gpx1*, we used translational blocking and splice blocking (sp) MOs. The animal poles of two cells staged developing *Xenopus* embryos were microinjected with 20-ng *gpx1* MO and 5-ng spMO to repress the *gpx1* expression. Microinjection of these MOs resulted in malformed eyes in injected embryos compared with the control MO-injected embryos (Figure 2A). It was observed that more than 70% of embryos showed malformed eyes after injection with translational blocking *gpx1,* while more than 50% of malformed embryos were observed after injecting with *gpx1* spMO (Figure 2B), although similar abnormalities were observed in both groups of embryos compared with the control embryos (Figure 2A). Furthermore, the malformed embryos were cryosectioned to observe the anatomical details of the eye, and the analysis of these sections showed incomplete detachment of the lens in *gpx1*-morphant embryos (Figure 2A).

To further elaborate the role of *gpx1* in eye development and to determine the specificity of *gpx1* MOs, rescue experiments were conducted using ebselen, a selenium-based compound that mimics glutathione peroxidases, with free-radical and singlet oxygen-quenching properties [29]. It catalyzes the reduction of hydrogen peroxide at the expense of thiols, just like glutathione peroxidases [29]. Ebselen was used for the rescue experiments because of the non-availability of *gpx1* specific antibodies in *X. laevis*. To rescue the malformed phenotypes induced by *gpx1* MOs, embryos were injected with *gpx1* MOs and then transferred to 0.5-μM ebselen solution. The ebselen-grown embryos recovered the malformations just like the control embryos, both in the case of translational blocking *gpx1* MO-injected embryos, as well as *gpx1* spMO-injected embryos (Figure 2C). Around 40–50% of embryos injected with *gpx1* MOs rescued the malformed eyes after being grown in ebselen (Figure 2D,E). Altogether, our data show the significance of *gpx1* during eye development and confirm the specificity of *gpx1* MOs.

To evaluate the significance of *prdx2* during *Xenopus* embryogenesis, 40 ng of *prdx2* MO and 1 ng of wild type *prdx2* mRNA were injected into two-cell stage *Xenopus* embryos. The *prdx2* MO injection did not induce any phenotypic malformations in morphant embryos and the *prdx2* morphant embryos were morphologically similar to the embryos injected with wild type *prdx2* mRNA, exhibiting no phenotypical malformations, just like the control embryos (Figure 2E). As the spatial expression of *prdx2* exhibited its expression in embryonic VBI, we speculated its involvement in embryonic blood cell development. To investigate the *prdx2* knockdown effect on embryonic blood cell development, we conducted a WISH analysis using blood cell markers, i.e., *hba3* (blood cell marker) and *mpo* (myeloid cell marker). The results of the WISH analysis showed that the expression of these markers was not affected by *prdx2* depletion, and similar expression and distribution of these markers were observed in the control embryos, *prdx2* morphant embryos, and embryos injected with *prdx2* mRNA (Figure 2F). Altogether, our results using *gpx1* and *prdx2* MO indicated the association of *gpx1* during embryogenesis, but no particular significance of *prdx2* was observed during *Xenopus* embryonic development.

### 3.3. *Gpx1* Interferes with Lens Development at the Molecular Level and Regulates ROS Levels in the Eye Regions during Embryogenesis

As the cross-sectional analysis of the eye in *gpx1*-morphant embryos revealed an incompletely detached lens compared to the control embryos in the case of both groups of MO-injected embryos, i.e., translational blocking *gpx1* MO and *gpx1* spMO (Figure 2A), to investigate the molecular details of *gpx1* knockdown during lens development, we performed WISH using the lens-specific marker crystalline beta A1 (*cryba1*) [30] to analyze the epithelial stem cells in the anterior lens pole.

**Figure 3:**
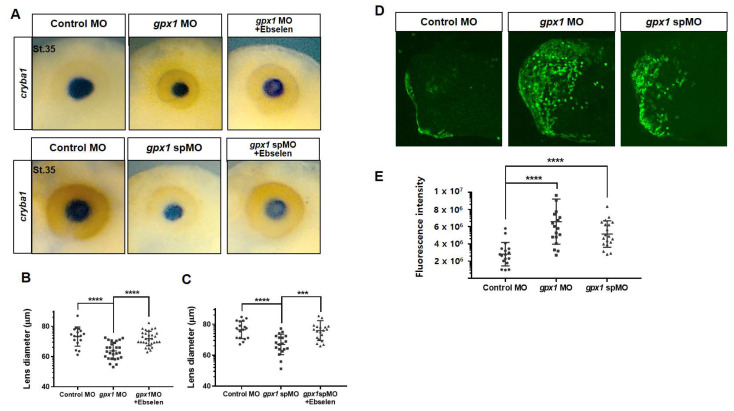
Loss of *gpx1* results in a perturbed expression of the lens-specific marker and an accumulation of ROS in developing eyes during *Xenopus* embryonic development. Here, (**A**) 20-ng *gpx1* MO and 5-ng *gpx1* spMO were microinjected into the animal pole of two-cell stage embryos, and the embryos were processed for WISH analysis using the lens-specific marker *cryba1*. The expression of *cryba1* was remarkably reduced in both groups of *gpx1*-morphant embryos. Moreover, the downregulated expression of *cryba1* was effectively rescued in *gpx1* morphants after ebselen treatment. (**B**) A graphical representation of *gpx1* MO-injected embryos revealed that *gpx1*-morphant embryos exhibited a lens diameter of 70 μm compared with >80 μm in the control embryos, and it was effectively recovered to 80 μm in the *gpx1*-depleted embryos after ebselen treatment. **** *p* ≤ 0.0001. (**C**) Statistical analysis of *gpx1* spMO-injected embryos indicated that *gpx1*-depleted embryos showed a lens diameter of approximately 75 μm as compared with >80 μm in the control embryos. The smaller size of the lens was effectively rescued to 80 μm after ebselen treatment in the *gpx1* spMO-injected embryos. *** *p* ≤ 0.001, **** *p* ≤ 0.0001. (**D**) Both *gpx1* MOs with HyPer mRNA (10 ng) were injected into the D.1.2. blastomeres of 16 cell stage *Xenopus* embryos and the *gpx1-*depleted embryos exhibited increased fluorescence intensity in the eye regions compared with the control embryos. (**E**) HyPer fluorescence intensity quantification clearly showed significant intense fluorescence in the *gpx1* MO and *gpx1* spMO injected embryos as compared with the control embryos. **** *p* ≤ 0.0001.

Our WISH analysis revealed that upon *gpx1* depletion by injecting both types of *gpx1* MOs (translational block and spMO), the *cryba1* expression was downregulated and small-sized lenses were observed in *gpx1*-morphant embryos (Figure 3A). The lens diameter was reduced to less than 70 μm in the *gpx1* MO-injected group and approximately 75 μm in the *gpx1* spMO-injected group compared with 80−85 μm in the control group (Figure 3B). To confirm that the smaller lens and downregulated expression of *cryba1* is particularly due to *gpx1* knockdown, we injected the embryos with *gpx1* MOs (translational block and splice block) and transferred the injected embryos to ebselen. The ebselen-grown embryos significantly rescued the *cryba1* expression and lens size, just like the control embryos (Figure 3A,B). Altogether, our results demonstrate that *gpx1* plays a significant role in eye development, affecting the lens during embryogenesis.

To assess the *gpx1* antioxidant function during eye development, we microinjected HyPer mRNA into the D.1.2 blastomere of 16 cell-staged embryos followed by *gpx1* MO injection (translational block and splice block). As expected, the fluorescence intensity was significantly increased in *gpx1* MO-injected embryos in the eye region compared with control embryos (Figure 3D). More intense fluorescence was observed in the translational block MO-injected group compared with the splice MO-injected group (Figure 3E). However, both groups showed a significantly higher intensity compared with the control group (Figure 3D,E). Our data clearly show that *gpx1* knockdown induced ROS accumulation in the eye region and evidently confirmed that the *gpx1* antioxidant activity is critical for embryonic eye development, along with its influence on specific eye markers.

### 3.4. *Gpx1* Regulates Cell Death and Interferes with Cell Migration during *Xenopus* Embryogenesis

**Figure 4:**
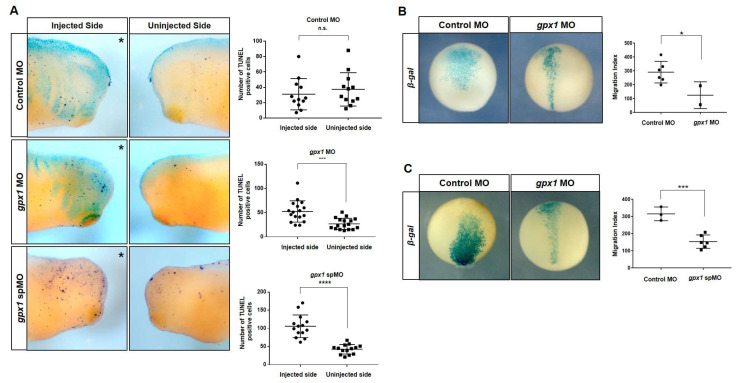
*gpx1* depletion activates cell apoptosis and inhibits cell proliferation, as well as affects cell migration during *Xenopus* embryogenesis. (**A**) The depletion of *gpx1* increased TUNEL-positive cells in the eye region of developing *gpx1* morphants compared with the control embryos. n.s. > 0.05, *** *p* ≤ 0.001, **** *p* ≤ 0.0001. (**B**) *gpx1* MO was coinjected with β-galactosidase mRNA into the D.1.2 blastomere of 16-cell stage *Xenopus* embryos. The staining showed that the cells were concentrated in the midline of the developing *gpx1*-depleted embryos compared with the widely distributed cells in the control embryos. The migration index was considerably reduced after *gpx1* MO injection. * *p* ≤ 0.05 (**C**) The injection of *gpx1* spMO into the D.1.2. blastomere also resulted in a cell concentration in the midline of developing embryos and decreased the migration index as compared with the control embryos. *** *p* ≤ 0.001.

Cell death plays significant roles in determining the organ size and particular organ structure. Different mechanisms maintain the proper size and structure of specific organs, such as apoptosis. Cell death is crucial in the development of the embryonic eye during embryogenesis. To elucidate whether eye malformations observed after *gpx1* knockdown involve the perturbation of cell death, a TUNEL assay was conducted. Our results of TUNEL staining indicated that TUNEL-positive cells were significantly increased on the *gpx1* MO injected side of *gpx1*-morphant embryos as compared with the uninjected side of the embryos (Figure 4A). The statistical analysis clearly showed the increase in TUNEL positive cells on the injected side of embryos as compared with the uninjected side of the embryos (Figure 4A), indicating the association of *gpx1* in cell death regulation.

To further investigate the effects of *gpx1* knockdown on cell migration during *X. laevis* embryogenesis, we microinjected β-galactosidase mRNA with *gpx1* MO and *gpx1* spMO into blastomere D.1.2. The results of this injection showed a broadened migration pattern for the control embryos, while for *gpx1* morphants, the cells were concentrated in the midline regions of developing embryos (Figure 4B). The migration index calculated by measuring the width of cells from the ventral to dorsal side clearly exhibited a decrease in migration as a result of *gpx1* depletion. The similar cell concentration in the midline of developing embryos was observed for *gpx1* spMO injected embryos and the migration index was considerably reduced as compared with the control embryos (Figure 4C). These results showed *gpx1* involvement in cell migration during early embryogenesis.

### 3.5. *Gpx1* Interferes with Wnt and Cadherin Signaling during Embryonic Eye Development in X. laevis

**Figure 5:**
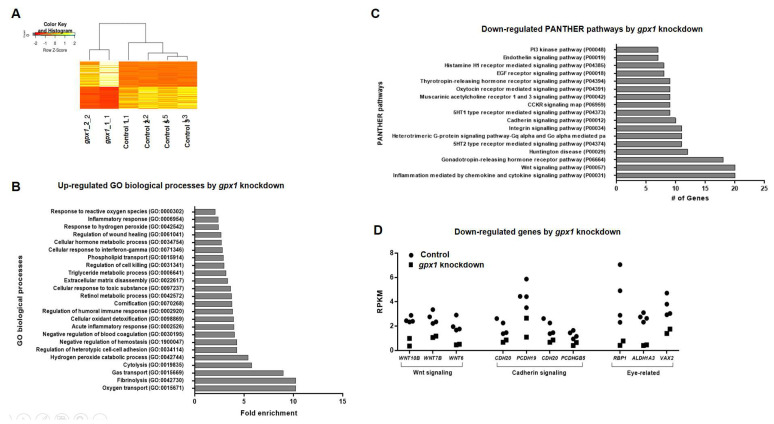
Transcriptomic analyses provide insight into the mechanism of eye development by *gpx1* during *Xenopus* embryogenesis. (**A**) The heat map shows the remarkable difference between the control embryos and *gpx1-*morphant embryos. (**B**) Transcriptome enrichment analysis using PANTHER software revealed the upregulation of the biological pathways associated with the response to reactive oxygen species, oxygen transport, hydrogen peroxide, hydrogen peroxide catabolic process, and gas transport. (**C**) Transcriptomic analysis using PANTHER software demonstrated the downregulation of signaling pathways, i.e., Wnt, Cadherin, and integrin associated with eye development in *gpx1*-depleted embryos. (**D**) Genes associated with Wnt signaling (Wnt10b, Wnt 7b, and Wnt6) showed significantly low RPKM values in *gpx1*-depleted embryos compared with the control embryos. A similar case was observed for cadherin signaling-associated genes (CDH20, PCDH19, and PCDHGB5), showing considerably low RPKM values compared with the control embryos. Additionally, genes associated with eye development, such as RBP1, ALDH1A3, and VAX2, exhibited remarkably low RPKM values after *gpx1* knockout compared with the control embryos.

To pinpoint the specific genes and associated pathways and to investigate the mechanism of eye development by *gpx1*, we conducted a transcriptomics analysis of *gpx1*-morphant embryos. The total RNA from *gpx1*-depleted embryos was extracted and processed for transcriptomics. Our transcriptomic analysis exhibited remarkable difference in the overall transcript expression between the control embryos and *gpx1*-morphant embryos (Figure 5A). *Gpx1* knockdown led to an upregulation of the pathways associated with oxygen transport, fibrinolysis, gas transport, and cytolysis, showing a fold-enrichment of approximately ten, while *gpx1* downregulation also induced the upregulation of the hydrogen peroxide catabolic process, cellular oxide detoxification, and response to hydrogen peroxide, but with a low fold-enrichment, i.e., less than five (Figure 5B). The most plausible reason for these upregulated biological responses associated with hydrogen peroxide is that ROS accumulation in *gpx1*-depleted embryos activated other antioxidant mechanisms to maintain a homeostatic balance [31]. In addition, depletion led to an upregulation of the cell killing (GO-0031341) pathway and it supported the increased number of TUNEL positive cells observed in *gpx1* morphant embryos (Figure 4A). Furthermore, the increased cell death after *gpx1* depletion was also supported by previously published data [32,33].

Because *gpx1* knockdown induced eye phenotypes and lens detachment, we analyzed the transcriptomic data for pathways associated with eye development, and our analysis revealed the downregulation of Wnt, cadherin, and integrin signaling pathways (Figure 5C); these three pathways are critical for eye organogenesis as well as in lens cell differentiation and lens development, and not the protection of the lens epithelial cell phenotype [34,35,36]. In the case of Wnt signaling pathway-associated genes, WNT10b, WNT7b, and WNT6 [37] showed RPKM values of <2 in *gpx1* morphants compared with the control embryos, showing RPKM values of approximately four (Figure 5D). Furthermore, genes associated with cadherin signaling, i.e., CDH20 and PCDHGB5 [38], exhibited RPKM values of less than two, whereas PCDH19 had RPKM values of less than three in the *gpx1*-depleted embryos as compared with the control embryos with RPKM values of >3 in CDH20 and PCDHGB5, and approximately 8 in PCDH19. In addition to the above genes associated with Wnt and cadherin signaling pathways, *gpx1* morphants exhibited a downregulation of RBP1 [39], ALDH1A3 [40], and VAX2 [41], with RPKM values of <2 compared with the control embryos showing RPKM values of ≥4 (Figure 5D).

The downregulation of the Wnt and cadherins pathways (Figure 5C) in *gpx1*-depleted embryos not only provide information about the eye phenotypes observed in *gpx1* morphant embryos, but also support the cell migration defects observed in *gpx1* morphant embryos (Figure 4B,C). Wnt and cadherin signaling play an essential role in vertebrate embryonic development by tightly controlling the cell migration, differentiation, and cell−cell adhesion [34,42].

Altogether, our transcriptomics data support *gpx1* involvement during embryogenesis, giving insight into the molecular pathways associated with embryonic eye development and indicating that *gpx1* knockdown led to interfering with the signaling pathways, which in turn led to defects in cell migration and differentiation. These cell migration defects might be the possible reason for the ocular abnormalities observed after *gpx1* depletion.

## 4. Discussion

Gpx1 and Prdx2 belonging to the thiol peroxidase class of antioxidants are the most abundant erythrocyte proteins to protect from oxidative damage. Both Gpx1 and Prdx2 have been studied for their antioxidant functions and have roles in different diseases [31]. Studies have implicated the role of Gpx1 in some cancers and cardiovascular diseases in humans [17,18,19,20]. Moreover, it is reported that Gpx1 is related to diabetes, and *Gpx1* overexpressed mice exhibited insulin resistance, hyperinsulinemia, obesity and hyperglycemia [43,44]. Prdx2 has a high expression level in various cancers, such as lung, colorectal, and gastric cancers [22]. Moreover, Prdx2 has been reported to be involved in arthrosclerosis progression [23]. However, the embryonic significance of Gpx1 and Prdx2 has not yet been elucidated. In this study, we sought to explore the physiological roles of Gpx1 and Prdx2 during *Xenopus* embryonic development.

*X. laevis* is an excellent model for the study of embryonic development, as the gene expression can be easily manipulated by RNA and DNA microinjection in fertilized eggs; subsequently, the gene expression can be analyzed through different stages of embryogenesis [27,45]. In this study, we systematically observed the spatiotemporal expression of *gpx1* and *prdx2* during *Xenopus* embryonic development. RT-PCR analysis revealed the zygotic nature of *gpx1* and the maternal nature of *prdx2* (Figure 1A,D). WISH analysis revealed *gpx1* localization in the eye regions, specifically in the lens of the developing embryos (Figure 1C), and prdx2 was localized in embryonic VB1 (Figure 1E). The functional significance of *gpx1* and *prdx2* during embryonic development was analyzed by microinjecting *gpx1* and *prdx2* into two-cell stage *Xenopus* embryos individually. Loss-of-function studies showed that *gpx1* is essential for eye development during embryogenesis. *Gpx1* morphants showed malformed eyes with incompletely detached lenses (Figure 2), indicating the critical roles of *gpx1* in embryonic eye development. However, *prdx2* depletion has no effect on developing embryos’ phenotypes, and *prdx2* morphant embryos appeared to be normal without any significant malformation (Figure 2E,F).

Embryonic development is a complicated process that relies on the interplay of different cellular processes and is regulated by various factors [46]. The intricate balance of ROS is a prerequisite for proper embryonic development [6]. Thus, we investigated the involvement of ROS in the abnormal eye phenotypes induced by *gpx1* knockdown. The HyPer mRNA injection in *Xenopus* embryos followed by *gpx1* MOs injection resulted in increased fluorescence intensity in *gpx1* morphants compared with the control embryos (Figure 3C). The increased fluorescence intensity in *gpx1* morphants supports ROS accumulation in the malformed eyes, which agrees with previous reports showing ROS involvement in poor eye development.

Eye development is a critical step in vertebrate embryogenesis that is initiated during gastrulation through the emergence of crescent-shaped eye fields in the anterior neural plate [47]. Embryonic eye development is regulated by different genes and transcriptiontional factors involved in lens cell differentiation and lens development, cornea and retinal formation, and finally proper eye development. As *gpx1* knockdown resulted in detached lenses in the morphant embryos, we investigated the expression of *cryba1* in *gpx1* morphants. The downregulated expression of *cryba1* and the decreased lens diameter (Figure 3A,B) in *gpx1* morphants indicated the interference of *gpx1* with molecular markers associated with lens development. Furthermore, the decreased lens diameter was supported by increased cell death in *gpx1*-depleted embryos (Figure 4A). The increased apoptotic cell death after *gpx1* depletion is in agreement with previously reported data [33]. These findings confirm that *gpx1* plays a critical role in eye development during *Xenopus* embryogenesis by regulating the cell growth and eye-related transcription factors.

The process of vertebrate eye development is regulated by different signaling pathways, and the malformed eyes observed in *gpx1* morphants may involve the perturbation of these signaling pathways [48]. The transcriptomic analysis revealed the downregulation of the Wnt, cadherin, and integrin signaling pathways. Wnt signaling plays an essential role in eye organogenesis and is active in the periocular surface ectoderm and lens epithelium during lens development [34,49]. *gpx1* knockdown resulted in the downregulation of Wnt signaling-associated genes, WNT10b, WNT7b, and WNT6 (Figure 5C,D), indicating the possibility of the involvement of *gpx1* in Wnt signaling.

Cadherin is significant for epithelial cell formation and retinal pigment epithelium is required for normal vision [35]. *gpx1* depletion resulted in the downregulation of the CDH20, PCDH19, and PCDHGB5 genes associated with cadherin signaling, showing the plausible association of *gpx1* with cadherin signaling (Figure 5C,D). Integrin plays an important role in lens development [36], and *gpx1* knockdown induced the downregulation of the integrin signaling pathway (Figure 5C). The downregulation of Wnt and cadherin signaling pathways supports the cell migration defects observed in *gpx1* depleted embryos. On the basis of transcriptomic analysis, we propose that *gpx1* interfered with Wnt and cadherin signaling pathways in developing embryos, which in turn led to the cell migration defects, and these migration defects ultimately led to ocular abnormalities in developing embryos.

## 5. Conclusions

Thiol peroxidases (Gpx1 and Prdx2) have essential antioxidant functions, but physiologically, *gpx1* is a prerequisite for normal embryonic development. Alternatively, *prdx2* does not appear to be associated with embryogenesis. The association of *gpx1* with lens-specific markers and key genes involved in eye development provides valuable information about the complex network involved in vertebrate eye development. *gpx1* perturbs normal eye development by regulating ROS levels, as well as Wnt, cadherin, and integrin signaling pathways. However, further detailed investigation is required to determine the regulatory pathways followed by *gpx1* to control embryonic eye development. To the best of our knowledge, this is the first study indicating the association of *gpx1* with embryogenesis, and it has emphasized the significance of antioxidants in embryonic development involving a complex network of signaling pathways.

## Data Availability

The data presented in this study are available in the manuscript.
